# Supplementary material for: A microfluidic device for inferring metabolic landscapes in yeast monolayer colonies
Source: eLife. 2019 Jul 1;8:e47951. doi: 10.7554/eLife.47951 (PMC6624017; doi:10.7554/eLife.47951)
Supplement: Supplementary file 1. [file elife-47951-supp1.docx]

# Supplementary File 1

| Name | Background | Genotype | Source |
| --- | --- | --- | --- |
| yPH001 | BY4741 | MATa his3Δ1 leu2Δ0 met15Δ0 ura3Δ0 | Leon’s Lab (IJM/ CNRS) |
| yPH152 | BY4741 | HXT1-GFP :HisMX | Leon’s Lab (IJM/ CNRS) |
| yPH155 | BY4741 | HXT7-GFP::HphNT | Leon’s Lab (IJM/ CNRS) |
| yPH179 | BY4741 | HXT2-GFP::HisMX | Yeast GFP collection |
| yPH180 | BY4741 | HXT3-GFP::HisMX | Yeast GFP collection |
| yPH182 | BY4741 | GLK1-GFP::HisMX | Yeast GFP collection |
| yPH183 | BY4741 | MIG1-GFP::HisMX | Yeast GFP collection |
| yPH188 | BY4741 | PDC1-GFP::HisMX | Yeast GFP collection |
| yPH189 | BY4741 | HXK1-GFP::HisMX | Yeast GFP collection |
| yPH190 | BY4741 | HXK2-GFP::HisMX | Yeast GFP collection |
| yPH191 | BY4741 | SDH2-GFP::HisMX | Yeast GFP collection |
| yPH192 | BY4741 | HXT4-GFP::HisMX | Leon’s Lab (IJM/ CNRS) |
| yPH193 | BY4741 | HXT5-GFP::HisMX | Leon’s Lab (IJM/ CNRS) |
| yPH236 | BY4741 | HXT6-GFP::Hyg | Leon’s Lab (IJM/ CNRS) |
